# Dihydropyrimidinase from *Saccharomyces kluyveri* can hydrolyse polyamides

**DOI:** 10.3389/fbioe.2023.1158226

**Published:** 2023-04-26

**Authors:** Felice Quartinello, Raditya Subagia, Sabine Zitzenbacher, Johanna Reich, Robert Vielnascher, Erik Becher, Mélanie Hall, Doris Ribitsch, Georg M. Guebitz

**Affiliations:** ^1^ Austrian Centre of Industrial Biotechnology, Tulln an der Donau, Austria; ^2^ Department of Agrobiotechnology, Institute of Environmental Biotechnology, IFA-Tulln, University of Natural Resources and Life Sciences, Vienna, Austria; ^3^ Institute of Chemistry, University of Graz, Graz, Austria; ^4^ BioHealth, University of Graz, Graz, Austria

**Keywords:** polyamide, hydrolysis, enzyme, surface functionalization, *Saccharomyces kluyveri*, dihydropyrimidinase

## Abstract

In *Saccharomyces kluyveri,* dihydropyrimidinase (DHPaseSK) is involved in the pyrimidine degradation pathway, which includes the reversible ring cleavage between nitrogen 3 and carbon 4 of 5,6-dihydrouracil. In this study, DPHaseSK was successfully cloned and expressed in *E. coli* BL-21 Gold (DE3) with and without affinity tags. Thereby, the Strep-tag enabled fastest purification and highest specific activity (9.5 ± 0.5 U/mg). The biochemically characterized DHPaseSK_Strep had similar kinetic parameters (K_cat_/K_m_) on 5,6-dihydrouracil (DHU) and *para*-nitroacetanilide respectively, with 7,229 and 4060 M^−1^ s^−1^. The hydrolytic ability of DHPaseSK_Strep to polyamides (PA) was tested on PA consisting of monomers with different chain length (PA-6, PA-6,6, PA-4,6, PA-4,10 and PA-12). According to LC-MS/TOF analysis, DHPaseSK_Strep showed a preference for films containing the shorter chain monomers (e.g., PA-4,6). In contrast, an amidase from *Nocardia farcinica* (NFpolyA) showed some preference for PA consisting of longer chain monomers. In conclusion, in this work DHPaseSK_Strep was demonstrated to be able to cleave amide bonds in synthetic polymers, which can be an important basis for development of functionalization and recycling processes for polyamide containing materials.

## 1 Introduction

Synthetic polymers are still heavily used in many industrial sectors. Although recycling rates are increasing and regulations are implemented, yet the amount of disposed material after their end of life is still tremendous. According to the report from Plastics Europe, most of the man-made polymers are landfilled, where these materials will be possibly fragmented into microplastics (Pellis et al., 2016; Europe and EPRO, 2019).

Consequently, innovative approaches are urgently required to recycle and valorize materials and to minimize their impact on our environment. Recovery of building blocks from polymers can involve chemical hydrolysis processes, where high concentrated acids or alkaline solutions are used (Yoshioka et al., 1994). Although the yield of such process is rather high, the whole procedure (including the neutralization steps, energy and required equipment) is quite expensive. A more environmentally friendly alternative approach is based on the exploitation of mild enzymatic hydrolysis to decompose polymers for the recovery of valuable monomers (Silva and Cavaco-Paulo, 2008; Wei and Zimmermann, 2017). Additionally, enzymes show a high specificity, thus allowing a step-wise recovery of pure building blocks even from a heterogenous mixture of composite materials (Quartinello et al., 2018; Kremser et al., 2021).

Enzymatic hydrolysis of man-made polymers has primarily been investigated and exploited for polyesters like poly (ethylene terephthalate) (PET) (Ribitsch et al., 2015; Gamerith et al., 2017). For example, the bacterium *Ideonella sakaiensis* (Yoshida et al., 2016) was reported to degrade and assimilate PET as well as different microbial communities, like the microbial consortium of rumen liquid from cows (Quartinello et al., 2021). These studies focused on biocatalysts belonging to the α/β-hydrolase family, including lipases, esterases and cutinases (Kallio et al., 2006), either for full decomposition into monomers or for surface functionalization. However, there are only few reports on enzymatic hydrolysis of polyurethanes (Gamerith et al., 2016; Bisceglie et al., 2022) or polyamides.

Polyamides are man-made polymers which can be synthetized from (a) an amino acid that undergo self-condensation or (b) by a ring-opening polymerization of the corresponding lactame or (c) a dicarboxylic acid and a diamine (Vol and Thermoplastics, 2003). These polymers present incredibly high strength, rigidity, chemical and thermal resistance. Polyamides (PA) are semicrystalline thermoplastics with outstanding mechanical properties and wide range of application. For example, PA-6 and PA-6,6 are well known and largely used in technical clothing and sportive apparel (Varghese and Grinstaff, 2022). PA with more than ten -CH_2_ groups between the amide bonds (e.g., PA-12) (Behler et al., 2007) have higher toughness and lower water adsorption. Generally, polyamides are produced from fossil-derived monomers, although in recent years there has been a new trend to produce polyamides based on castor oil (e.g., PA-11, Rilsan^®^) (Menezes et al., 2022) and/or derived from putrescine (butane-1,4-diamine) or cadaverine (pentane-1,4-diamine). The latter is nowadays largely produced in *Escherichia coli* (Lee et al., 2020).

The potential of enzymes including cutinases, amidases and proteases (Parvinzadeh, 2009) for the hydrolysis of amide bonds in polyamide has been investigated both for surface functionalization (e.g., to improve wettability) as well as related to complete decomposition (Song and Kim, 2017) (22). Amidases from *Nocardia farcinica* and *Beauveria brongniartii* have been examined for their ability to hydrolyze PA oligomers and fibers (Almansa et al., 2008; Heumann et al., 2009). More recently, genetic engineering was used to tune specificity cutinases which are extensively described for polyester hydrolysis to hydrolyse amide bonds (Biundo et al., 2019). The *Humicola insolens* cutinase, for instance, was site directed mutated in order to relocate the water network and generate hydrogen bonds with a substrate containing amide bonds. In addition to hydrolases, various oxidative enzymes have been proven to depolymerize polyamides. Fujisawa et al. (Fujisawa et al., 2002) demonstrated that applying a manganese peroxidase in presence of 1-hydroxybenzotriazole (HBT) as mediator, the surface of polyamide-6 and polyamide-6,6 was modified without size reduction of the fibers.

**FIGURE 1:**
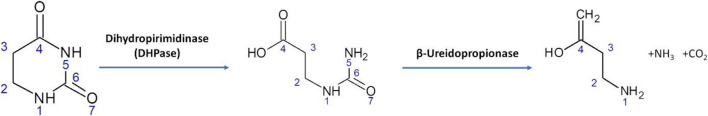
Reductive degradation pathway for uracil catalyzed by DHPase from *Saccharomyces kluyveri*.

Dihydropyrimidinases (DHPase), also known as dihydropyriminidine amidohydrolases, catalyze the ring opening of 5,6-dihydrouracil to N-carbamyl-β-alanine and 5,6-dihydrothimine to N-carbamyl-β-amino isobutyrate, which represents the second of three steps of the reductive degradation pathways for the pyrimidine bases uracyl and thymine. This ring cleavage is a reversible reaction, which leads the opening of the ring between nitrogen 3 and carbon 4 (Figure 1) (Beck et al., 2008).

Thus, DHPase activity is highly required for the regulation of the available pyrimidine pool content either in yeast or mammalians. The deficiency of this catalytic activity is well known in mammalians: due its crucial role in the metabolic pathway, DHPase, deficiency is defined as dihydropyrimidinuria (Nyhan, 2005). It is a rare disease where the phenotype manifests seizure, mental and growth retardation and dysmorphic features. DHPase is also involved in the metabolism of various drugs. For example, it works as antioxidative agent against the side effects of doxorubicin, a chemotherapy drug used against colon and breast cancer (Huang et al., 2019).

Due many different genetic and/or medical implications, various DHPases (from yeasts to *Drosophila melanogaster*) have been purified from their natural sources or produced as recombinant protein. Independently from the organisms, DPHase is a homotetrameric Zinc^2+^-metalloenzyme, where the subunit molecular weight can range from 56 to 65 kDa, which are mainly interconnected by hydrophobic interactions and to a lesser extent by hydrogen bonds. The subunit of DHPase consists of a catalytic core domain and a smaller *β*-sandwich domain. The core is composed of a (β/α)8-barrel structure. Further, the catalytic di-zinc center is embedded in this structure and accompanied by the *β*-sandwich domain (Lohkamp et al., 2006).

This study focuses on the investigation of a so far unknown activity of *Saccharomyces kluyveri* dihydropyrimidinase (DHPaseSK) towards various structurally different polyamide films. A detailed characterization of the (poly)amide hydrolysis mechanism was carried out using Liquid Chromatography-Time of Flight/Mass Spectrometry (LC-MS/TOF) and Fourier-Transformed Infrared Spectroscopy (FT-IR).

## 2 Materials and methods

### 2.1 Chemicals and reagents

Buffer components and model substrates such 5,6-dihydrouracil (DHU) and *para*-nitroacetanilide (pNAA) were purchased from Sigma-Aldrich (Vienna, Austria). All other chemicals and reagents used in this work were of analytical grade and used without further purification if not otherwise specified. LB media premix for culturing *Escherichia coli* BL21-Gold (DE3) was purchased from Roth, Germany. The following chemicals (Sigma-Aldrich, Germany) were dissolved in Milli-Q water and sterile filtered: 50 mg mL^−1^ kanamycin, 1 M ZnCl_2_ and 1 M IPTG.

### 2.2 Cloning, expression, and purification of DHPaseSK

A codon optimized version of the gene encoding *S. kluyveri* dihydropyrimidinase (DHPaseSK, NCBI accession number Q9P903.1) was used to synthesize a variant without affinity tag (DHPaseSK), a variant with N-terminal His-Tag (DHPaseSK_His) and a variant with N-terminal Strep-Tag II (DHPaseSK_Strep) (GenScript, Piscataway, NJ, USA). The gene was cloned downstream of the T7 promotor into the *E. coli* expression vector pET26b (+) over the NdeI and HindIII restriction sites.

The chemically competent *E. coli* BL21-Gold (DE3) cells (Invitrogen, Carlsbad, CA, USA) were transformed with each variant via heat shock, and the freshly transformed cells, starting from a single colony, were cultured overnight in LB media containing 40 μg/mL kanamycin at 37°C, 150 rpm (HT Multitron Pro, Infors, Bottmingen, Schweiz). The overnight culture was used to inoculate the main culture, starting with an optical density of 0.1 measured at wavelength of 600 nm (OD_600_) in 300 mL auto induction media (AIM) containing 40 μg/mL kanamycin in 1 L shake flask. AIM, consisting of 6 g/L Na_2_HPO_4_, 3 g/L KH_2_PO_4_, 29 g/L tryptone/peptone, 5 g/L yeast extract, and 5 g/L NaCl, was adjusted to pH 7.2. After sterilization of afore mentioned components, sterile glycerol 0.6% (w/v), glucose 0.05% (w/v) and lactose 0.2% (w/v) were added completing the auto induction media. After reaching OD_600_ of 1 at 37 C 160 rpm, 50 μM ZnCl_2_ was added to the main culture, and incubated further at 20 C for 48 h. The protein expression was induced by the lactose metabolism after depletion of glucose in the medium.

After harvesting the cells at 3,400 g for 20 min, the cells were resuspended in the corresponding binding buffer with a ratio of 5 mL binding buffer per 1 g pellet. Extraction of the DHPaseSK expressed in cytoplasmic space took place by disruption of the resuspended cell pellet through sonication (Digital sonifier, Branson, CT, United States), applying 10 rounds x 10 s pulses of 60% amplitude with 2 min rest between rounds. The lysate was centrifuged at 20,000 g for 25 min, and sterile filtrated (PES 0.22 µm) before loaded (1 mL/min) onto the corresponding column using the Äkta system (Äkta pure, GE Healthcare, Chalfont St. Giles, United Kingdom). The column was washed with binding buffer (1 mL/min) until the baseline was reached, removing most of the unspecific bound impurities. Subsequently, the enzyme was eluted during a 15-column volume gradient from 0% to 100% of elution buffer.

Following columns and buffers were used for the different variants: DHPaseSK without Tag having a theoretical pI of 5.1 was purified via anion exchange chromatography, using Hitrap Q XL 1 mL (Cytiva, Marlborough, MA, United States), the binding buffer: 20 mM Tris HCl pH 7.6), and the elution buffer: 20 mM Tris HCl pH 7.6, 1 M NaCl. The His tagged enzyme DHPaseSK_His was purified via affinity chromatography using a 5 mL HisTrap FF (Cytiva, Marlborough, MA, USA), the binding buffer: 20 mM Na_2_HPO_4_.* 2 H_2_O, 500 mM NaCl, 10 mM Imidazol pH 7.4, and the elution buffer: 20 mM Na_2_HPO_4_.*2 H_2_O, 500 mM NaCl, 500 mM Imidazol pH 7.4. The Strep-tagged enzyme DHPaseSK_Strep was purified over the Strep-Tactin Superflow column (IBA, Göttingen, Germany) using the binding buffer: 100 mM Tris/HCl pH 8.0, 150 mM NaCl and the elution buffer: 100 mM Tris/HCl pH 8.0, 150 mM NaCl, 50 mM biotin respectively.

The eluted fractions were pooled and concentrated using centrifugal concentrator, vivaspin 20 with 10 kDa molecular cut off (Sartorius Göttingen, Deutschland). The buffer was exchanged to 100 mM Tris/HCl pH 7 using PD-10 desalting columns (GE Healthcare, Chalfont St. Giles, United Kingdom) and the enzyme was stored at −20°C until further use.

DHPaseSK expression levels and enzyme purities were analyzed by SDS PAGE and the protein concentrations after purification was determined with Bradford protein assay. The protein concentration was determined with the Biorad protein assay (Bio-Rad laboratories GmbH, Vienna, Austria) as previously described (Perz et al., 2016).

### 2.3 Biochemical characterization of DHPaseSK

Hydrolysis of the 5,6- dihydrouracil (DHU) (final concentration 1 mM) ring leads to decrease in absorbance. The molar absorption coefficient for DHU at 225 nm is 1.28 mmol^−1^ cm^−1^ (KAUTZ and SCHNACKERZ, 1989); this change was measured at 225 nm with Tecan INFINITE M200 plate reader (Männedorf, Switzerland) (Turan and Sinan, 2005).

The optimal buffer and pH value were determined at 25 C with DHU over a pH range of 6–9, using the following buffer systems: sodium phosphate buffer (pH 6–8), potassium phosphate buffer (pH 6–8), and Tris-HCl buffer (pH 7–9). In parallel different ionic concentrations of the tested buffer were used (100-250-500-1,000 mM). Moreover, the same assays were performed in presence of 2 mM ZnCl_2_, in order to determine the possible influence of the ion to the enzymatic activity. The role of ZnCl_2_ was tested in presence of EDTA. Briefly, to the enzyme solution (in the optimal buffer) were added 1 or 2 mM of EDTA prior to ZnCl_2_, and then afterwards the activity was measured as previously described.

In order to determine the optimal temperature, the enzyme solution was incubated for 15 min at different temperatures in the range of 25°C–85 C with the identified optimal buffer conditions and then the activity assay with 5,6-dihydrouracil was performed as previously described.

To investigate the thermal stability in the range of the optimal temperature, the enzyme was incubated for up to 72 h at different temperatures, in the range of 45°C–55 C. Samples were collected at the time points as indicated and the residual activity was measured according to the standard assay.

### 2.4 Kinetics of DHPaseSK

The Michaelis-Menten parameters K_m_ and k_cat_ were determined using DHU and pNAA in a concentration range of 0.5–5 mM at 25 C. The parameters were calculated by simple weighted non-linear regression of the Michaelis-Menten equation using the SigmaPlot 11.0/Software—Enzyme Kinetics 1.3 (Systat Software GmbH, Erkrath, Germany).

The increase in the absorbance at 385 nm (for pNAA) was measured using a Tecan INFINITE M200 plate reader (Männedorf, Switzerland). A blank was measured using 20 μL of buffer instead of sample. The increase in the absorbance (at 25 C) indicated an increase p-nitroaniline (ε_385 nm_ = 9.25 mmol^−1^ cm^−1^). The activity was calculated in units, where 1 unit had been defined as being the amount of enzyme required to hydrolyze 1 μmol of substrate per minute under the given assay condition.

### 2.5 Hydrolysis of polyamides

Hydrolysis of different polyamides, namely, PA-6, PA-6,6, PA-4,6, PA-4,10 and PA-12 (chemical structures in Supplementary Figure S1) by DHPaseSK was investigated. The different films (with size 1*0.5 cm) were primarily washed (Vecchiato et al., 2017) and then incubated in triplicate in 2 mL of the so-defined optimal buffer conditions containing 5 µM of DHPaseSK. The solution was incubated at 55°C and 150 rpm (identified as optimal temperature). Samples were collected at the following time points: 1, 24, 48, 72, 120 and 168 h.

The amidase from *Nocardia farcinica* (NFpolyA) has previously demonstrated polyamidase activity on PA oligomers (Heumann et al., 2009). Therefore, activities of DHPaseSK and this enzyme likewise dosed at 5 µM pure protein were compared.

### 2.6 Liquid-chromatography mass spectroscopy/time-of-flight (LC-MS/TOF)

A total of 2 mL of each sample was centrifuged at 12,700 rpm and 4 C for 15 min and the supernatant was filtered in PTFE filters with a cut-off of 0.2 μm. Liquid chromatography coupled to a time-of-flight/mass spectrometer (LC-MS/TOF) in positive ionization mode was used to qualitatively identify the released soluble oligomers. The analytes were separated using HPLC (1,260 series, Agilent Technologies, Palo Alto, CA, United States) equipped with a reversed-phase C18 rapid resolution column (Zorbax Eclipse XDB, Agilent Technologies, Palo Alto, CA, United States) of 50 mm by 2.1 mm and 1.8 µm particle diameter at a total runtime of 15 min. Column temperature was set to 40 C. Mobile phase A consisted of 20 mM NH_4_COOH in ultrapure water and mobile phase B was MS-grade acetonitrile.

The flow rate was 0.5 mL min^−1^ and the injection volume was 20 µL. This HPLC system was connected to a time-of-flight mass spectrometer (G6230B, Agilent Technologies, Palo Alto, CA, United States) equipped with a dual electrospray ionizer (ESI) under the following operating conditions: capillary 3500 V, nebulizer 40 psig, drying gas 8 L min^−1^, gas temperature 300°C, fragmentor 125 V, skimmer 65 V, OCT 1 RF Vpp 750 V. The mass axis was calibrated using the mixture provided by Agilent Technologies over the mass range of 50–3,200 m/z. Spectra were acquired with the Agilent Technologies software Mass Hunter (Version 10.1) over the 50–3,000 m/z range at a scan rate of two spectra per second (Bisceglie et al., 2022). In Supplementary Table S1 are listed the monomers, dimers and trimers detected and their respective molecular weight.

### 2.7 FT-IR analysis

The film samples were washed with 5 g/L Triton X-100, 100 mM Na_2_CO_3_, 3 M urea and ultrapure water sequentially for 30 min for each solution to remove the possible adsorbed enzymes, salt residues and other impurities from their surface. The samples were then dried at room temperature for 24 h and the spectra were recorded on a PerkinElmer Spectrum 100 spectrometer. Spectra were collected at a resolution of 2 cm^−1^ for 48 scans and normalized at the area of 2,300–2,200 cm^−1^ before data processing (Quartinello et al., 2019).

## 3 Results and discussion

### 3.1 Expression and purification of dihydropyrimidinase from *Saccharomyces kluyvery*

In the past, DHPase from S. *kluyveri* was cloned and expressed as His8-tagged protein in *E. coli* for structural elucidation (Lohkamp et al., 2006). However, analysis of the three-dimensional structure revealed that the enzyme comprises an (β/α)_8_-barrel structural core embedding a catalytic di-zinc center that can potentially be leached out by a His-Tag. To prevent such effects and to enable the production of a fully Zn^2+^-loaded and active enzyme, DHPaseSK was also produced without Tag as well as well as with a C-terminal Strep-Tag. All three codon-optimized versions of *S. kluyveri* dihydropyrimidinase, namely, DHPaseSK_His, DHPaseSK and DHPaseSK_Strep were overexpressed in *E. coli* at 20 C in presence of 50 µM ZnCl_2_, providing sufficient zinc ions for complete incorporation. Although ZnCl_2_ showed precipitation after being added to the media, the dissolution improved after agitation. Besides, protein expression via auto induction media has been chosen over induction by IPTG to yield sufficient amounts of soluble DHPaseSK.

**FIGURE 2:**
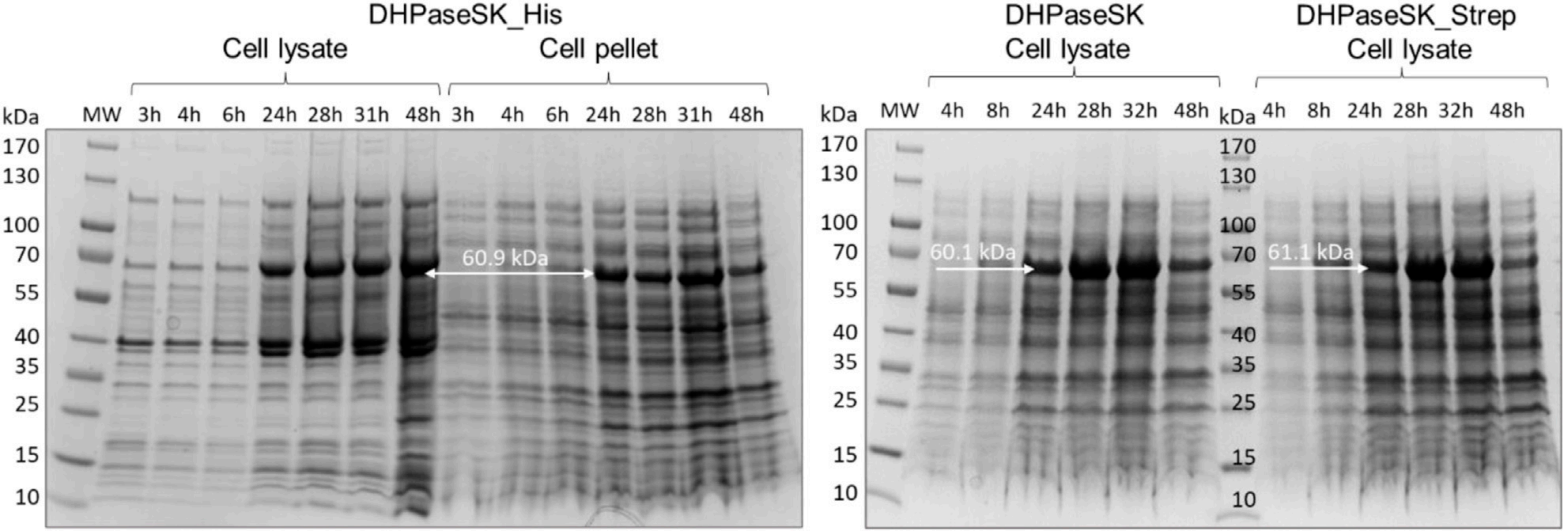
SDS-PAGE of dihydropyrimidinase from *Saccharomyces kluyvery* (DHPaseSK) variants produced in *Escherichia coli*. Samples were withdrawn after 3–48 h of auto induction at 20 C. Expected molecular masses are 60.1 kDa (DHPaseSK), 61.1 kDa (DHPaseSK_Strep) and 60.9 kDa (DHPaseSK_His).

As shown in Figure 2, all three enzyme variants were expressed to a comparable extent in soluble form at 60.9 kDa (DHPaseSK_His), 60.1 kDa (DHPaseSK) and 61.1 kDa (DHPaseSK_Strep) with respective purity of 69.7, 57.1% and 82.7% (Supplementary Figure S2). However, all variants also showed a significant level of insoluble enzyme, as presented by DHPaseSK_Strep. Interestingly, DHPaseSK_His reveals additional protein bands around 40 kDa which could result from degraded enzyme.

Even if the expression of the variants was comparable, the determination of the specific activities towards DHU revealed large differences. Untagged DHPaseSK showed 9.5 ± 0.5 U/mg under the standard assay conditions of 25°C and pH 7. Variant DHPaseSK_Strep showed higher activity with 10.5 ± 0.3 U/mg whereas DHPaseSK_His revealed only 4.3 ± 0.3 U/mg. The results clearly demonstrate that the His-Tag negatively affects activity. Due to the faster purification procedure by the use of the affinity Tag and the higher activity towards DHU, it was decided to carry out further investigations of the enzyme with the Strep-tagged variant DHPase_Strep.

### 3.2 Biochemical characterization and kinetics of DHPaseSK_Strep

**FIGURE 3:**
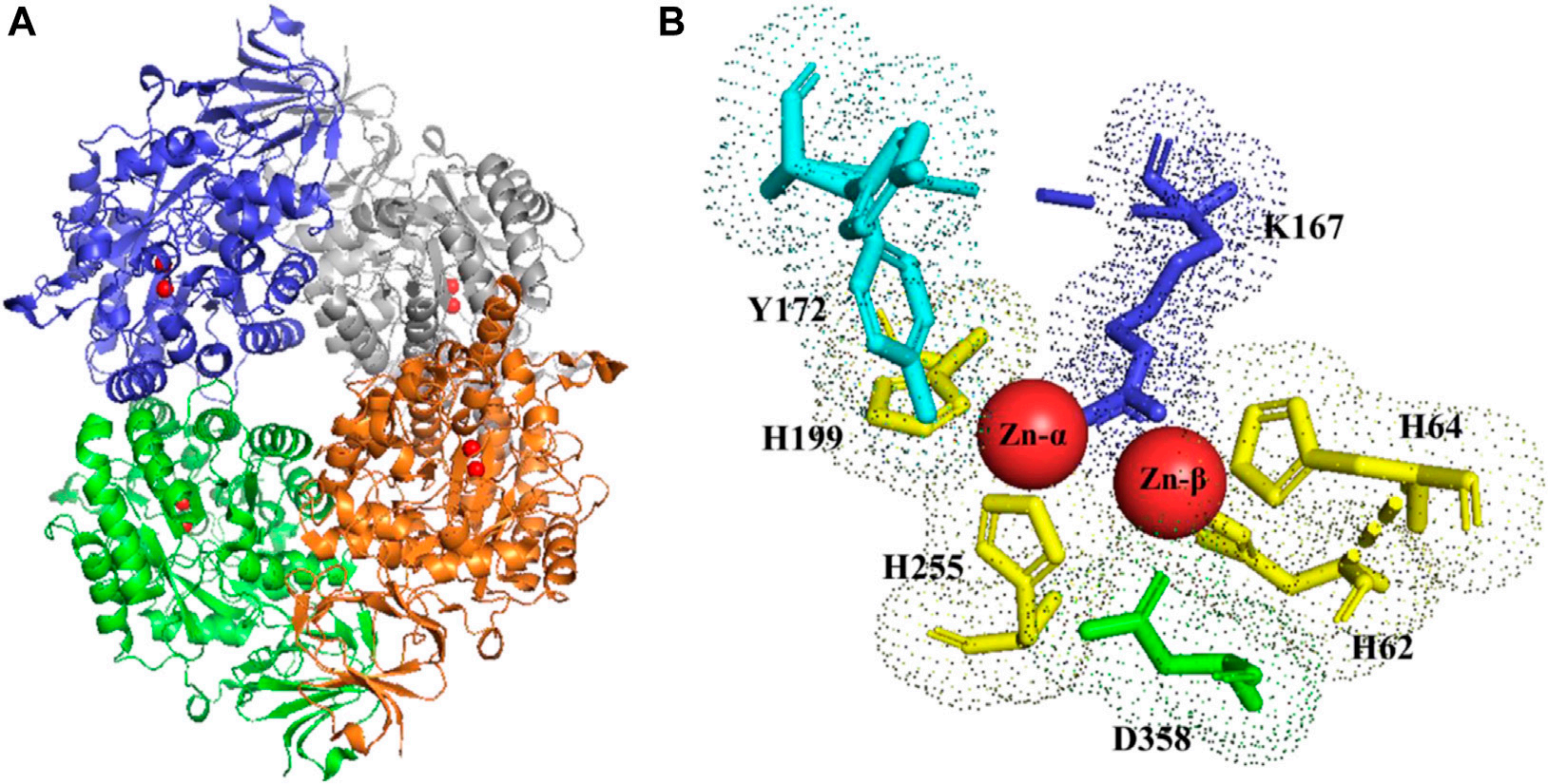
DHPase **(A)** tetrameric structure (zinc ions highlighted in red). **(B)** stereo view of the di-zinc center and ligand binding aminoacids of DHPase. Y172 (cyano); K167 (blue); H 62-64-199-255 (yellow); D358 (green). PDB structure (2FTY) (Huang et al., 2019)

It is generally assumed that the reaction mechanism of DHPase is similar to the proposed mechanism of dihydroorotase (DHOase) (Guan et al., 2021). The Zn-ß, as the cofactor of the prosthetic group of DHPase, can activate a water molecule, which is placed in the coordination sphere of the metal ion. For activation, respectively for deprotonation of the zinc-bridging hydroxyl ion, Asp^358^/Asp^325^ act as base which are also able to ligate Zn^2+^(Figure 3).

In case of DHU as substrate for hydrolysis, the described water molecule of the more solvent and exposed Zn-ß is replaced by the 4-oxo group of the incoming substrate, leading to polarization of the carbonyl group. In consequence, nucleophilic attack of the zinc bridging hydroxyl ion on the C-4 atom of DHU leads to formation of a geminal diol. As a result of the nucleophilic addition, the substrate and the prosthetic group build a tetrahedral intermediate. The ring cleavage is mediated by protonation from Asp^358^, leading to the breaking of the covalent C-N bond. Finally, the product N-carbamyl ß-alanine (NCßA) is released. In order to regenerate the active side, a new water molecule is bound and activated (Lohkamp et al., 2006).

**FIGURE 4:**
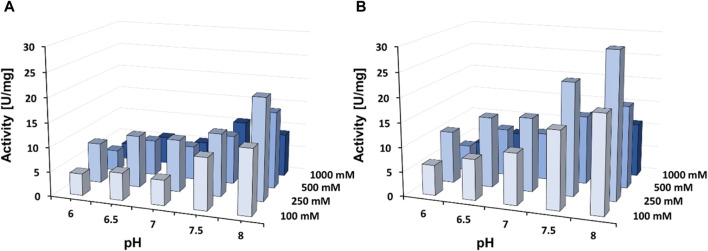
Enzyme activity of the dihydropyrimidinase from *Saccharomyces kluyvery* DHPaseSK_Strep towards DHU in presence of 2 mM ZnCl_2_ at 25°C with **(A)** without of 2 mM ZnCl_2_ **(B)** in presence of 2 mM ZnCl_2_. Tests were performed in triplicates.

Purified DHPaseSK_Strep was tested for its activity towards DHU in presence of three different buffer systems, in combination with different ionic strengths. Sodium phosphate and Tris HCl buffer demonstrated to be the less efficient systems of the enzyme of interest (Supplementary Figure S3). In detail, in presence of potassium phosphate, the enzyme activity seemed to increase between pH 7.5 and 8.0. On the other hand, the increase of the ionic strength had a negative effect on DHPaseSK_Strep activity. The maximum activity value was given in using 250 mM concentration of buffer, especially with potassium phosphate pH 8. Besides, the addition of zinc also influences the activity strongly. Addition of 2 mM ZnCl_2_ improved the DHPaseSK_Strep activity of the previous optimal buffer condition around 55% (31.3 U/mg in presence of Zn against 20.1 U/mg without zinc ion) (Figure 4). These buffer system conditions are comparable to other purified DHPases already characterized from the Persian silk tree, *Albizzia julibrissin* (Turan and Sinan, 2005). Upon addition of EDTA in the buffer system, the DHPaseSK activity was drastically reduced, confirming the crucial role of these cations for the enzymes.

**FIGURE 5:**
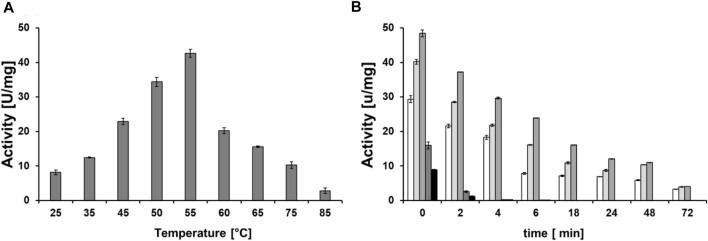
**(A)** Temperature optimum of DHPaseSK_Strep. **(B)** Thermal stability of the dihydropyrimidinase from *Saccharomyces kluyvery* DHPaseSK using DHU as substrate. Tests were performed in triplicates.

Once the optimal buffer condition had been defined, DHPaseSK_Strep optimal temperature and thermal stability were determined. Following the biocatalytic activity with DHU, the activity increased from 25°C to 55°C, where the maximum activity was established. At temperature >55°C, the activity decreased strongly (Figure 5A). At this temperature the enzyme showed also highest thermal stability. In detail, after 3 days of incubation, the enzyme was still active, although the activity decreased by 80%. At temperature of 60°C and 65°C, no activity was detected after 4 h of incubation. At lower temperature than 55°C, the activity was lower but still detectable with the full incubation assay (Figure 5B). Considering these results, 55°C was then used for the polyamide-hydrolytic activity experiments.

The kinetic parameters of the enzyme were determined against the substrate DHU (specific substrate) and against p-nitroacetanilide (pNAA) as PA model substrate. In Table 1, it is shown that substrate pNAA is as well hydrolyzed even though it is not the natural substrate of DHPaseSK. Although the chemical structure of the substrates is dissimilar, the biocatalysts was able to cleave the amide bonds from both, while NFpolyA demonstrated only activity on *p*-NAA.

**TABLE 1:** Kinetic parameters of the dihydropyrimidinase from *Saccharomyces kluyvery* DHPaseSK_Strep.

	Kcat/K_m_ (M^−1^ s^−1^)	
Substrate	DHPaseSK	NFpolyA
Dihydrouracil (DHU)	7,229	n.d
*p*-nitroacetanilide (pNAA)	4,060	5,125

### 3.3 Activity of DHPaseSK_Strep on polyamides

**FIGURE 6:**
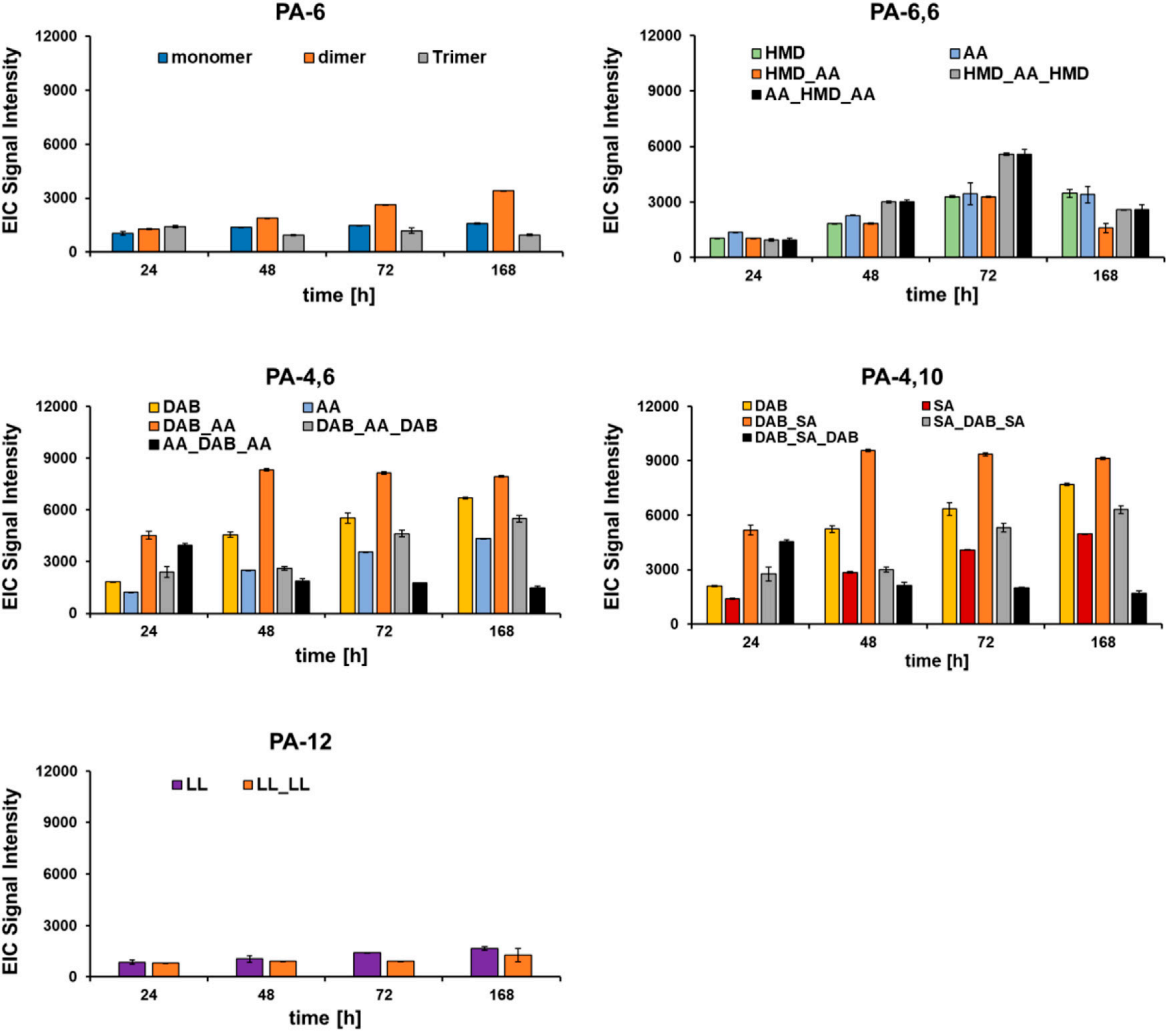
Hydrolysis of different polyamides by DHPaseSK. Hydrolysis products were analyzed by using LC-MS/TOF. Test were performed in triplicates. HMD: hexamethylenediamine; AA: adipic acid; DAB: 1,4-diamine butane; SA: sebacic acid; LL: laurolactame.

Once the optimal hydrolysis conditions had been evaluated on model substrates, the DHPaseSK_Strep was incubated with PA films of different chemical composition (PA-6, PA-6,6, PA-4,6, PA-4,10 and PA-12) and compared to the *Nocardia farcinica* amidase. Despite the low homologies of these enzymes (Supplementary Figure S3), both enzymes were able to hydrolyze these polyamides based on LC-MS/TOF analysis of released monomers, dimers and trimer. In detail, from PA6, in a first phase the enzymes released the corresponding trimer which was successively further cleaved. However, accumulation of the dimer indicates slower hydrolysis into monomer when compared to the larger trimer (Figure 6; Supplementary Figure S4). Accumulation of the dimers was likewise seen for PA4,6 and for PA 4,10.

For PA-6,6, the corresponding trimers, namely, AA_HMB_AA and HMB_AA_HMB were found in similar amounts upon hydrolysis with DHPaseSK_Strep while during hydrolysis with the enzymes from *N. farcinica* AA_HMB_AA seems to accumulate. This might indicate a different preference of the individual enzymes to bind and hydrolyze either oligomeric diamines or oligomeric diacids. On the other hand, PA-6 after incubation with DHPaseSK led to a lower extent of released monomer and oligomers, compared to PA-6,6.

Comparing the hydrolysis results from PA-6,6 and PA-4,6, similar amounts of the monomer adipic acid were released after 168 h. In comparison, from PA4,6 and from PA4,10 higher amounts of the diamine DAB were found from DHPaseSK_Strep, while for the enzymes *N. farcinica* higher amounts of the corresponding acids were detected.

This reduction is probably due to the fact that this trimer was initially produced as products of oligomer hydrolysis and were then progressively hydrolyzed, allowing the release of DAB. These results indicated some preference of DHPaseSK_Strep to hydrolyze amide bonds between shorter chains substrates while the *N. farcinica* amidase released higher amounts from longer chain polymides.

**FIGURE 7:**
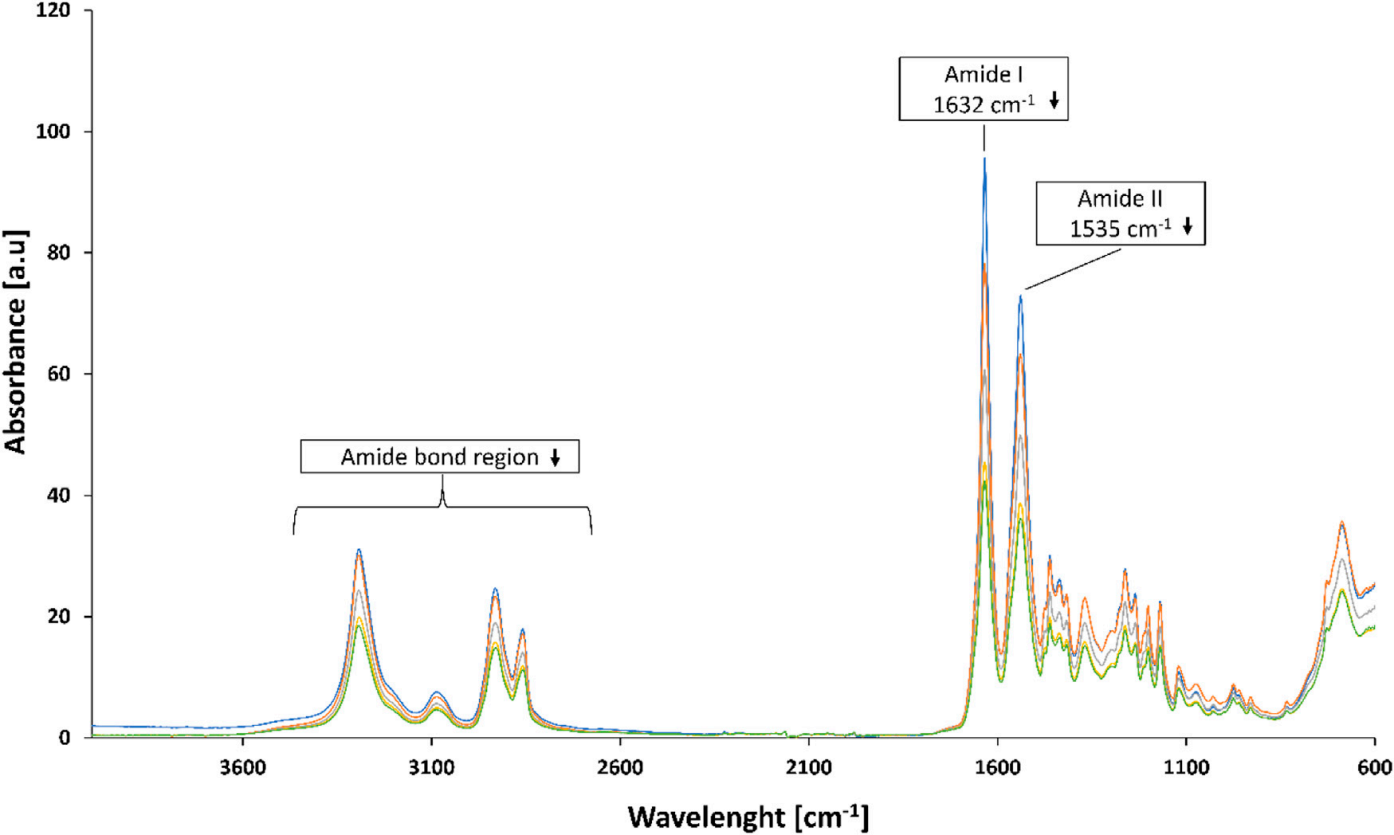
FT-IR analysis of PA-4,6 before and after hydrolysis by DHPaseSK for 0 h (blue line), 24 h (orange line), 48 h (grey line), 72 h d (yellow line) and 168 h (green line).

FTIR analysis of PA-4,6 hydrolysed by *DHPaseSK* (Figure 7) indicated differences in the 3,300–2,800 cm^−1^ region and reduction of the amide I (1,632 cm^−1^) and amide II (1,535 cm^−1^) signals at FT-IR are indicative of the cleavage of amide bond (Figure 4). FT-IR spectra of the other polyamides did not show any significant changes upon enzymatic hydrolysis (Supplementary Figures S5—S8).

## 4 Conclusion and outlook

In conclusion, in the present work, a dihydropirimidinase from *Saccharomyces kluyvery* was successfully expressed and purified. The DHPaseSK variant purified with Strep-tag showed higher purity and activity towards DHU similar to the untagged enzyme as well as activity on pNAA. This enzyme was then biochemically characterized in terms of pH, ionic strength, optimal temperature and thermal stability. The enzyme activity was positively influenced in the presence of zinc.

In this study, we demonstrated for the first-time hydrolysis activity of a dihydropirimidinase on polyamides. DHPaseSK_Strep hydrolyzed various polyamides with some preference to short chain substrates monomer like PA-4,6. Further studies should involve larger scale production of the DHPaseSK_Strep to allow investigation of decomposition of industrial polyamides based materials, multilayer and blended materials as well as wastes.

## Data Availability

The original contributions presented in the study are included in the article/Supplementary Material, further inquiries can be directed to the corresponding author.
