# Supplementary material for: Dihydropyrimidinase from Saccharomyces kluyveri can hydrolyse polyamides
Source: Front Bioeng Biotechnol. 2023 Apr 26;11:1158226. doi: 10.3389/fbioe.2023.1158226 (PMC10169691; doi:10.3389/fbioe.2023.1158226)
Supplement: Supplementary file 1 [file DataSheet1.PDF]

Supplementary Material

Dihydropyrimidinase from *Saccharomyces kluyveri* can hydrolyse polyamide

Felice Quartinello[1,2], Raditya Subagia[1], Johanna Reich[1], Robert Vielnascher[1], Erik Becher[3], Mélanie Hall[3,4], Doris Ribitsch[1,2*], Georg M. Guebitz[1,2]

[1] Austrian Centre of Industrial Biotechnology, Konrad Lorenz Strasse 20, 3430 Tulln an der Donau, Austria

[2] Department of Agrobiotechnology IFA-Tulln, Institute of Environmental Biotechnology, University of Natural Resources and Life Sciences, Vienna, Austria

[3] Institute of Chemistry, University of Graz, Heinrichstrasse 28, 8010 Graz, Austria

[4] BioHealth, University of Graz, Austria

[*]Corresponding author: Doris Ribitsch Mag. Dr, tel: +43 1 47654-97487, email: doris.ribitsch@boku.ac.at

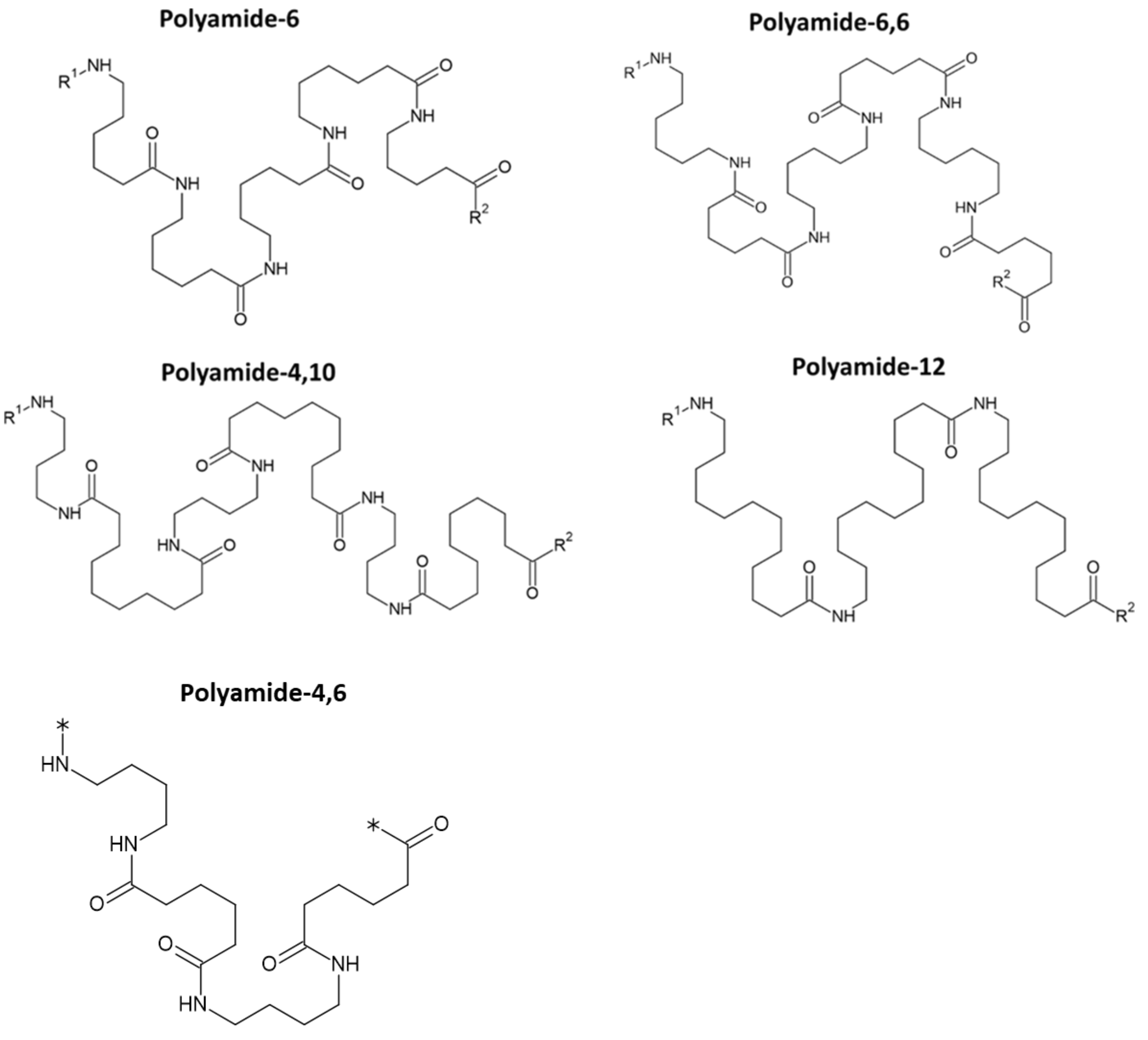

Figure 1 Nylon structures

Table 1 List of monomers, dimers and trimers potentially released during enzymatic hydrolysis of structurally different polyamides detected via LC-MS/ToF and their relative molecular weighs

Polymer	Monomer 1	Monomer 2	Dimer	Trimer 1	Trimer 2
PA-6	$C_6H_{11}NO$<br>M_W =115		M_W =246	M_W =360	
PA-6,6	Hexamethilenediamine<br>(HMD) $C_6H_{16}N_2$<br>M_W =116.21	Adipic acid<br>(AA) $C_6H_{10}O_4$<br>M_W =146	M_W =246	HMD_AA_HMD<br>1 AA<br>M_W =344	AA_HMD_AA<br>M_W =326

PA-4,6	1,4 diaminebutane<br>(DAB) $C_4H_{12}N_2$<br>M_W =89	Adipic acid<br>(AA) $C_6H_{10}O_4$<br>M_W =146	M_W =217	DAB_AA_DAB<br><br>M_W =290	AA_DAB_AA<br>M_W =346
PA-4,10	1,4 diaminebutane<br>(DAB) $C_4H_{12}N_2$<br>M_W =89	Sebacic acid<br>(SA) $C_{10}H_{18}O_4$<br>M_W =203	M_W =274	SA_DAB_SA<br>2 SA<br>M_W =434	DAB_SA_DAB<br>M_W =346
PA-12	Laurolactame<br>(LL) C12H_{23}NO<br>M_W =198		M_W =414	M_W =614	

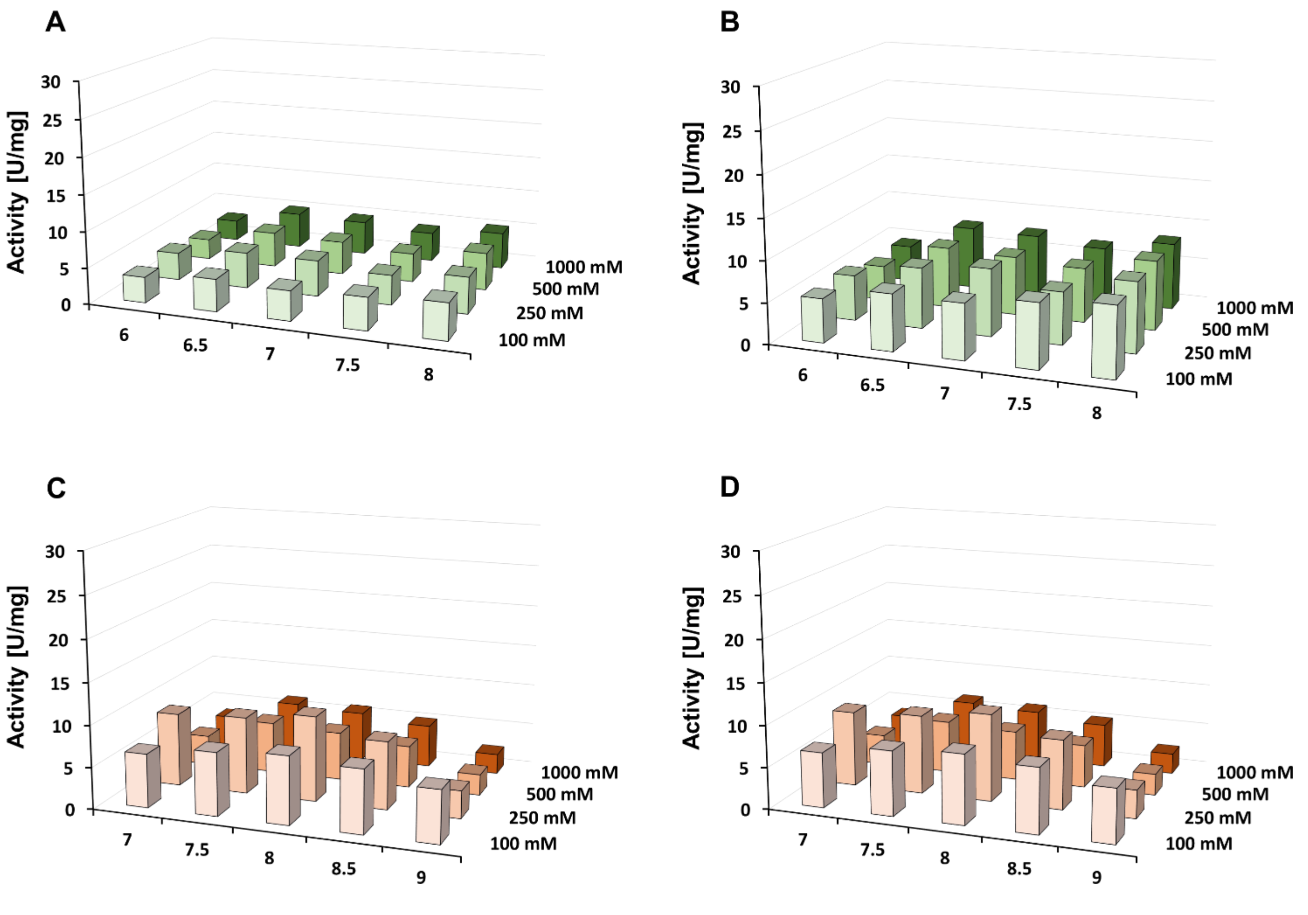

Figure 2 Enzyme activity of DHPaseSK_Strep at 25 °C towards DHU without $ZnCl_2$ by using a) Sodium phosphate b) potassium phosphate c) Tris-HCl

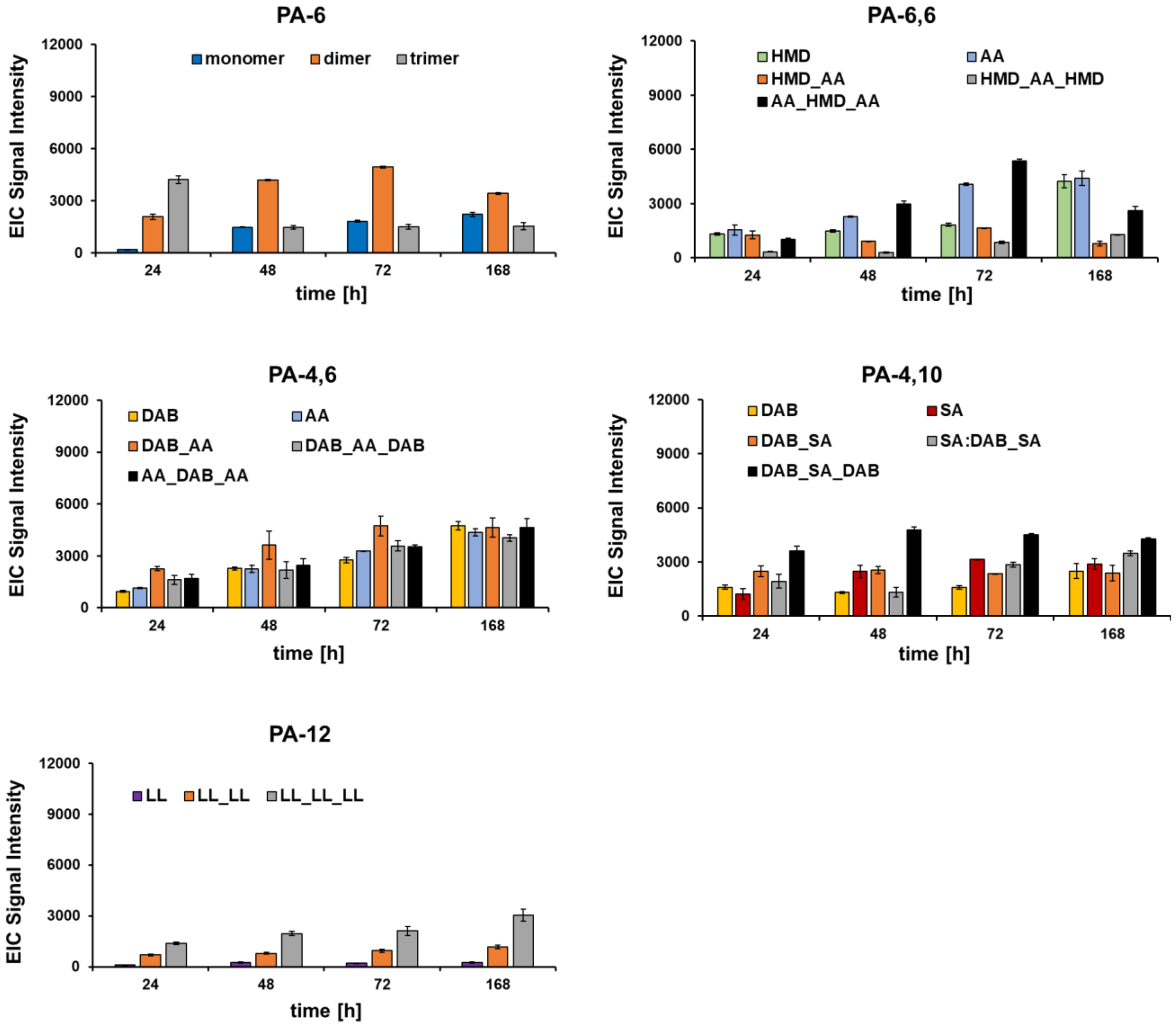

Figure 3 Hydrolysis of different polyamides by Nocardia farcinica amidase. Hydrolysis products were analyzed by using LC-MS/TOF. Test were performed in triplicates. HMD: hexamethylenediamine; AA: adipic acid; DAB: 1,4-diamine butane;SA: sebacic acid; LL: laurolactame

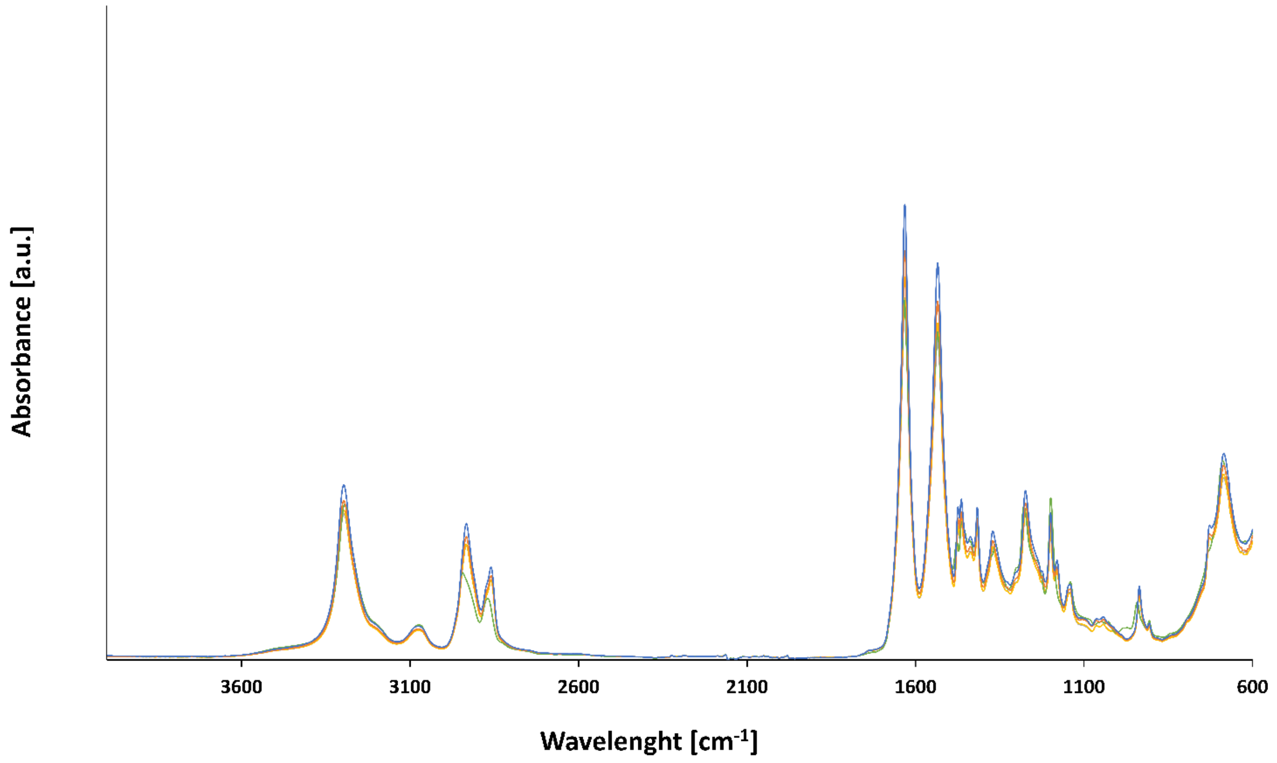

Figure 4 FT-IR analysis of PA-4,10 before and after hydrolysis by DHPaseSK for 0 hrs (blue line), 24 h (orange line), 48 h (grey line), 72 h d (yellow line) and 168 h (green line)

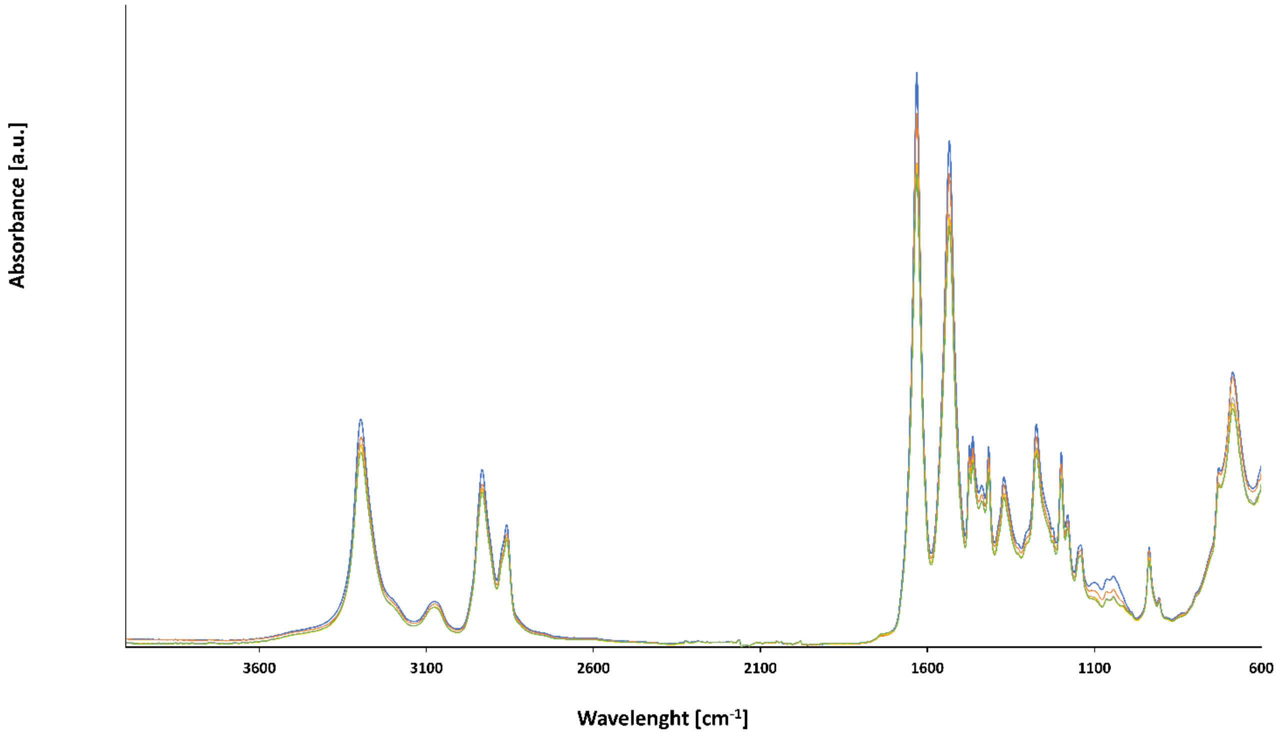

Figure 5 FT-IR analysis of PA-6,6 before and after hydrolysis by DHPaseSK for 0 hrs (blue line), 24 h (orange line), 48 h (grey line), 72 h d (yellow line) and 168 h (green line)

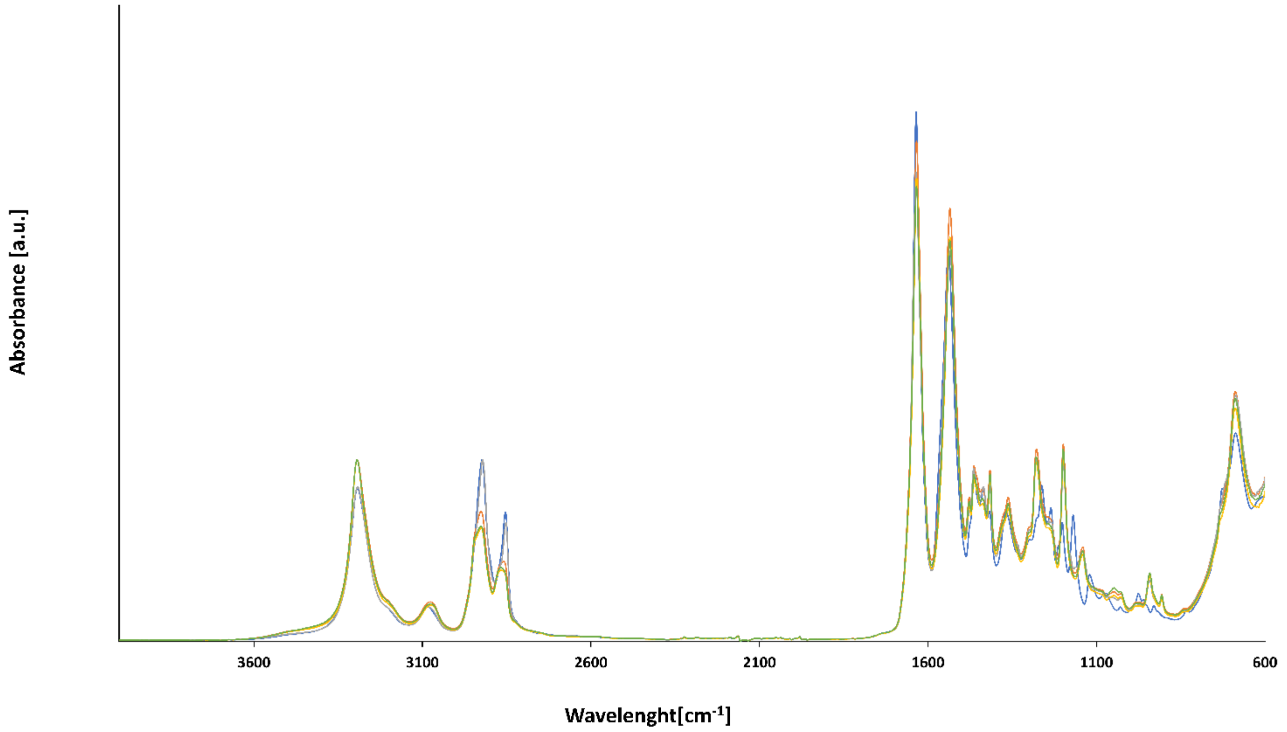

Figure 6 FT-IR analysis of PA-6 before and after hydrolysis by DHPaseSK for 0 hrs (blue line), 24 h (orange line), 48 h (grey line), 72 h d (yellow line) and 168 h (green line)

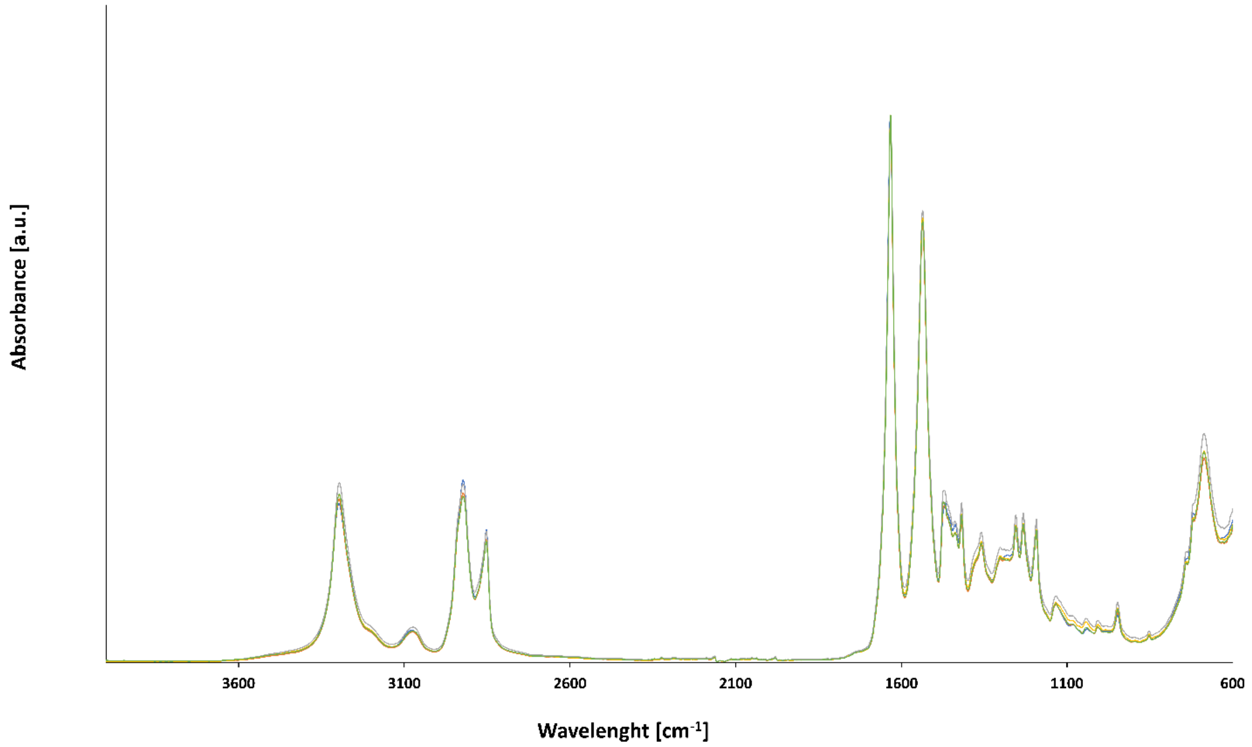

Figure 7 FT-IR analysis of PA-12 before and after hydrolysis by DHPaseSK for 0 hrs (blue line), 24 h (orange line), 48 h (grey line), 72 h d (yellow line) and 168 h (green line)
